# Cost-Outcome Descriptive Study for Mobile App (UPSCALER) in the Rehabilitation of Anterior Cruciate Ligament (ACL) Injuries After Reconstructive Surgery

**DOI:** 10.7759/cureus.59886

**Published:** 2024-05-08

**Authors:** Eng Kee Tan, Khairil Anwar Ahmad Hanif, Firdati Mohamed Saaid, Raymond D.K. Yeak, Johan Abdul Kahar, Aidalina Mahmud, Nizlan M Nasir

**Affiliations:** 1 Department of Orthopedics and Traumatology, Universiti Putra Malaysia, Serdang, MYS; 2 Department of Community Health, Universiti Putra Malaysia, Serdang, MYS

**Keywords:** cost-outcome, mobile application, acl reconstruction, accelerated regime, acl rehabilitation

## Abstract

Anterior cruciate ligament (ACL) injuries are a significant concern in athletes, often leading to long-term complications and reduced quality of life. Despite advancements in surgical techniques, outcomes following ACL reconstruction remain suboptimal, largely due to poor adherence to postoperative rehabilitation. This study introduces a novel postoperative rehabilitation approach utilizing a smartphone application, UPSCALER, developed by the Universiti Putra Malaysia Sports Injury and Arthroscopic Surgery Center of Excellence. The application delivers a validated accelerated rehabilitation protocol through instructional videos tailored to each patient's recovery phase. Results from the study demonstrate promising outcomes, including improvements in Knee Injury and Osteoarthritis Outcome Score (KOOS) subscales post-rehabilitation, potentially attributed to increased adherence facilitated by the application's accessibility. Furthermore, the study explores the cost-effectiveness of this approach compared to conventional methods. In conclusion, smartphone application-guided rehabilitation shows promise in improving ACL reconstruction outcomes, warranting further research to validate its effectiveness and long-term impact on patient recovery and healthcare costs.

## Introduction

Anterior cruciate ligament (ACL) injuries are a common and significant form of injury seen in athletes. The consequences and complications associated with this injury can be devastating, leading to a delayed return to sports, long-term functional knee deficiencies, reduced quality of life, financial burdens on the patients and healthcare system, as well as early onset osteoarthritis [1]. Despite numerous advances in surgical techniques, outcomes after ACL reconstruction surgeries are still considered to be poor. As low as 55% of patients who undergo ACL reconstruction are able to return to their pre-morbid status and return to sport [1]. Patients often report a reduced knee functional status and are burdened with less than satisfactory results in the postoperative period. Multiple studies have demonstrated that postoperative rehabilitation is an extremely crucial component in the holistic approach to managing a patient with an ACL injury [2-5]. There has also been a paradigm shift from the more traditional rehabilitation program towards a more accelerated regime. Despite this, the outcomes after ACL reconstruction are still less than desirable. One reason for this is adherence or compliance with the rehabilitation regime. It is hypothesized that the usage of a smartphone application as the platform for delivering rehabilitation guidance will improve the adherence of patients to the regime, and thus improve postoperative outcomes.

Much research has been performed to advance the surgical technique and technology involved in ACL reconstruction surgery. There have also been numerous publications that compare the efficacy and effectiveness of the accelerated rehabilitation regime against the conventional regime. Numerous authors have also identified factors that influence adherence and subsequently, the patient’s outcome after surgery and rehabilitation [6-9]. However, the final outcome remains unfavorable and there has been little evidence to demonstrate a reliable method of improving patient adherence to rehabilitation.

This paper analyzes a novel form of information provision to patients undergoing ACL reconstruction surgery as a guide for postoperative rehabilitation and evaluates the cost versus outcome. It utilizes a smartphone application designed by the Universiti Putra Malaysia (UPM) Sports Injury and Arthroscopic Surgery Center of Excellence (SIASCOE) using a validated accelerated regime as its guide.

This study was conducted in a Malaysian university teaching hospital over a period of one year. Similar studies have not been performed as this method of guiding postoperative rehabilitation is still a new development.

## Materials and methods

Setting

This was a prospective study conducted over the period of one year at the Sultan Abdul Aziz Shah Hospital (HSAAS) Universiti Putra Malaysia (UPM). The Ethics Committee for Research Involving Human Subjects, UPM issued approval (JKEUPM-2022-294). Consenting patients above the age of 18 from both genders who underwent elective arthroscopic ACL reconstruction surgery were included in this study. Five patients were recruited into this study, aged between 24 and 38. Two were male, three were female, and all completed tertiary education. Of the five ACL grafts performed, two were hamstring, and three were bone-patella tendon-bone grafts.

UPSCALER app

The Universiti Putra SIASCOE ACL Exercises & Rehabilitation (UPSCALER) app was fully designed and coded by the UPM SIASCOE team (see Appendices). It was designed purely for information provision purposes where the patients were able to obtain videos to guide their rehabilitation regime. The rehabilitation process was divided into different phases which were subsequently unlocked by the consulting physician during follow-up once the patient was assessed and deemed fit to proceed. This prevented patients from pre-emptively participating in a more advanced phase, which may lead to complications.

All information within the app was based on the accelerated protocol for post-ACL reconstruction rehabilitation (reference). No new treatment methods were being tested, and the purpose of this study was purely to determine the feasibility and effectiveness of the UPSCALER app as a means to provide information and guided rehabilitation.

Knee Injury and Osteoarthritis Outcome Score (KOOS)

This aided in determining the efficacy and effectiveness of the rehabilitation regime. Factors influencing the outcome can also be identified in order to determine predictors of outcome and prognosis [2]. The consensus statement by the ACL Consensus Meeting Panther Symposium in 2019 stated that in ACL outcome studies, assessment of patient-reported outcomes should optimally include a knee-specific outcome tool, an activity rating scale, and a measure of quality-of-life. The Knee Injury and Osteoarthritis Outcome Score (KOOS) is a patient-administered questionnaire with five subscales that cover these recommended criteria. These subscales are pain, knee symptoms, activities of daily living (ADL), sports, and quality of life (QoL).

KOOS published in 1998 is a widely used tool for research and clinical purposes for monitoring patients over time. It is intended for use in patients with knee injuries that can result in post-traumatic osteoarthritis, for example, ACL injuries. Its content validity was based on the literature review, a pilot study, and an expert panel from the United States and Sweden consisting of patients, orthopedic surgeons, and physical therapists [10,11]. The assessment is divided into five subscales and is patient-administered. It is available in the public domain free of charge, and no licensing or permission is required for usage.

## Results

The startup cost involved the development of the application. Software development was done by a member of the SIASCOE team. An independent graphics designer was engaged for the entire duration of the development phase. Coding was done using a personal computer with a browser-based coding software provided free by the Massachusetts Institute of Technology (MIT) called the MIT App Inventor. Videography was performed by the same SIASCOE member who performed the coding. Cost also involved a one-time developer account fee with the Android Google Play Store. Startup costing is listed in Table 1.

**Table 1:** Startup costing for the UPSCALER app. UPSCALER: Universiti Putra SIASCOE (Sports Injury and Arthroscopic Surgery Center of Excellence) ACL (Anterior Cruciate Ligament) Exercises & Rehabilitation; USD: United States dollar.

Item	Cost
Coding	Nil
Graphics designer	USD 1000
Coding computer	USD 1500
Coding software	Nil
Videography equipment	USD 2000
Developer account	USD 25

The cost of providing this service during the implementation phase was calculated based on the number of visits to the physiotherapist and clinic follow-up during the rehabilitation period.

During each visit, the patient would undergo registration, followed by consultation with either the physiotherapist or the treating surgeon.

In this study, patients were followed up in clinics by the treating surgeon at the end of each phase (two weeks, six weeks, 10 weeks, and six months postoperatively). These visits coincided with physiotherapy appointments.

Human resource costs for clinic and physiotherapy visits are calculated as (salary of staff X time used to attend to the patient) at the registration counter and for consultation during both clinic and physiotherapy visits. Cost per visit (CPV) is calculated based on the staff’s salary per week, averaged into hourly wages, and then calculated based on the time spent with the patient for each event. Cost per visit is then multiplied by four (a total of four visits over six months), for clinic and physiotherapy visits respectively to obtain the total cost per patient (CPP). This is presented in Table 2.

**Table 2:** Breakdown of cost per patient. CPV: Cost per visit; CPP: Cost per patient; USD: United States dollar.

Event	Staff salary/week	Time spent on patient	CPV	CPP
Clinic registration	USD 250	10 minutes	USD 1.04	USD 4.16
Clinic consultation	USD 600	15 minutes	USD 3.75	USD 15
Physiotherapy registration	USD 250	10 minutes	USD 1.04	USD 4.16
Physiotherapy consultation	USD 400	1 hour	USD 10	USD 40
			Total CPP	USD 63.32

Five patients were enrolled in this study. The outcome was measured using the KOOS scoring system before and after intervention. Table 3 shows the mean scores across all subscales of the KOOS scoring system and the difference in scores before and after the rehabilitation regime. The score difference is then used to calculate the cost per point for each subscale.

**Table 3:** Mean KOOS scores and cost per point for each subscale. KOOS: Knee Injury and Osteoarthritis Outcome Score; ADL: Activities of daily living; QoL: Quality of life; USD: United States dollar.

	Mean			
KOOS subscale	Pre-rehab	Post-rehab	Difference	Cost per point
Pain	79.5	94.5	15	USD 4.22
Symptoms	77	82.25	5.25	USD 12.06
ADL	78	85	7	USD 9.05
Sports	50	87.5	37.5	USD 1.69
QoL	31.5	79.75	48.25	USD 1.31

## Discussion

One of the main factors for a poor outcome after ACL reconstruction is non-adherence to rehabilitation [2]. Multiple publications have discussed the role of psychological factors in the outcome after surgery in ACL injuries. Grindem et al. summarized the rehabilitation process as time-consuming, placing a high demand on the patient’s mental and emotional state [3]. They have also found that patients are not necessarily motivated during the rehabilitation process and suggested that motivation can be obtained via patient education, goal-setting, and regular feedback [3,4]. However, the rehabilitation process tends to focus on the physical aspects, without much emphasis on the psychological factors experienced by the patient despite evidence to the contrary whereby goal-setting and adequate support have been found to increase adherence rates and improve outcomes. Therefore, it is recommended that practitioners enhance the patient’s self-efficacy and motivation [5,8,12]. This is supported by Brewer et al. who found that self-motivation is a significant predictor of adherence to the rehabilitation regime [6]. With regard to self-efficacy and motivation, Walker et al. as well as Everhart et al. state that strategies that enhance these factors have an influence in obtaining positive outcomes while emphasizing the value of the advancements in digital health and the role they may play [1,9,13]. Higher adherence is also associated with greater independence of the patient, and they rely less on the physiotherapist’s input [14,15]. These factors are especially significant in the accelerated protocol as it requires a combination of home and clinic-based regimes, placing a heavier burden on the patient in terms of adherence to the regime despite home-based regimes being equally effective compared to standard clinic-based regimes [7,16,17]. Therefore, optimizing rehabilitation has become a necessity as patients tend to discontinue their rehabilitation three months after surgery [18,19]. A systematic review by Risberg et al. compared home-based versus supervised rehabilitation regimes [20]. They found that there was no significant difference between the two methods, indicating that home-based programs can be just as effective while being more cost-efficient for the patient and healthcare provider [20].

There is a need to introduce and implement regimes that enhance recovery in order to achieve pre-morbid stability and function after surgery [21,22]. One of the commonest persistent deficiencies associated with ACL injuries after surgery is quadriceps weakness and its strength deficit can exceed 20% even six months after surgery [23,24]. While the ACL reconstruction surgery aims to restore knee stability, restoration of joint function is achieved via exercise regimes during the rehabilitation period [25,26]. In order to combat the issue of adherence and expedite return to activity, the accelerated postoperative ACL reconstruction rehabilitation protocol was developed and largely adopted. A study by De Carlo et al. revealed that patients who underwent the accelerated regime fared better than those in the traditional regime, achieving a full range of motions and full quadriceps strength earlier, without compromising on knee stability [27,28]. Multiple publications have described the accelerated regime and its phases, spanning over 6 months with clear goals for each phase [29-32]. While the information provided in the smartphone application was adopted from these publications for the purpose of this study, the method of information provision to the patient, together with a tracking system within the application were likely the reasons for good compliance and an improvement in KOOS subscale scores.

There have been numerous studies conducted on the cost-effectiveness of different ACL treatment methods. Mather et al. compared the incremental cost-effectiveness ratio (ICER) between early versus delayed reconstruction in ACL injury patients and found the average cost to be USD 21454 in the delayed reconstruction group, while the early reconstruction group had a cost of USD 19883 [33]. This translated to an ICER of USD 4434 versus USD 3881 per quality-adjusted life-year (QALY) in the delayed reconstruction (DR) and early reconstruction (ER) groups respectively [33]. This is supported further in a systematic review by Deviandri et al., demonstrating a higher quality-adjusted life year (QALY) gained per patient [34].

In an effort to reduce the cost of healthcare to the patient, there has been increased advocacy towards home-based rehabilitation regimes. Outcome comparisons between supervised and home-based regimes have shown similar results, and in some studies, the home-based regimes were found to be superior while having better cost-effectiveness [35]. Grant et al. demonstrated this in a Level 1 Evidence Randomized Clinical Trial in 2005, with Grant and Mohtadi further confirming it in a two- to four-year follow-up conducted in 2010 [36,37].

To the authors’ knowledge, this study is the first that has attempted to estimate the cost involved in an Android-based application-guided rehabilitation regime for ACL reconstruction patients. Direct comparison of savings generated with this new method is difficult. Despite this, current literature supports the implementation of home-based rehabilitation regimes, and this novel method of information provision to guide rehabilitation would further enable patients to conduct their rehabilitation in the comforts of their own homes, while potentially improving compliance and reducing cost.

This study was limited by its small sample size due to its selection criteria. With a sample size of five patients, it may not represent the broader population. A larger sample size would improve the significance of the study and its data. This could also be achieved by performing a multi-center study involving a control group in order to enable a direct comparison between the treatment and control arms, as well as aid in confirming the reproducibility of results. A multi-center study would also include a more diverse patient population instead of confining the study to a single university hospital.

## Conclusions

Anterior cruciate ligament (ACL) injuries pose significant challenges to athletes, often resulting in long-term functional deficits and reduced quality of life. Despite advancements in surgical techniques, outcomes following ACL reconstruction remain suboptimal, with poor adherence to postoperative rehabilitation being a key contributing factor. This study introduces a novel approach to postoperative rehabilitation using a smartphone application, UPSCALER, designed to guide patients through their rehabilitation process. The application, developed by the Universiti Putra Malaysia (UPM) Sports Injury and Arthroscopic Surgery Center of Excellence (SIASCOE), utilizes an accelerated rehabilitation protocol and provides patients with instructional videos tailored to their recovery phase. Results from the study indicate promising outcomes, with improvements observed in Knee Injury and Osteoarthritis Outcome Score (KOOS) subscale scores post-rehabilitation. Notably, patients demonstrated increased adherence to the rehabilitation regime, potentially attributed to the convenience and accessibility offered by the smartphone application. Furthermore, the study explores the cost-effectiveness of implementing a smartphone application-guided rehabilitation regime compared to conventional methods. While direct comparisons are challenging, the study provides valuable insights into the potential savings and benefits associated with this innovative approach.

In conclusion, the utilization of smartphone applications in guiding postoperative ACL reconstruction rehabilitation represents a promising avenue for improving patient outcomes. Further research and larger-scale studies are warranted to validate these findings and explore the long-term impact of such interventions on patient recovery and healthcare costs.

## Figures and Tables

**Figure 1:**
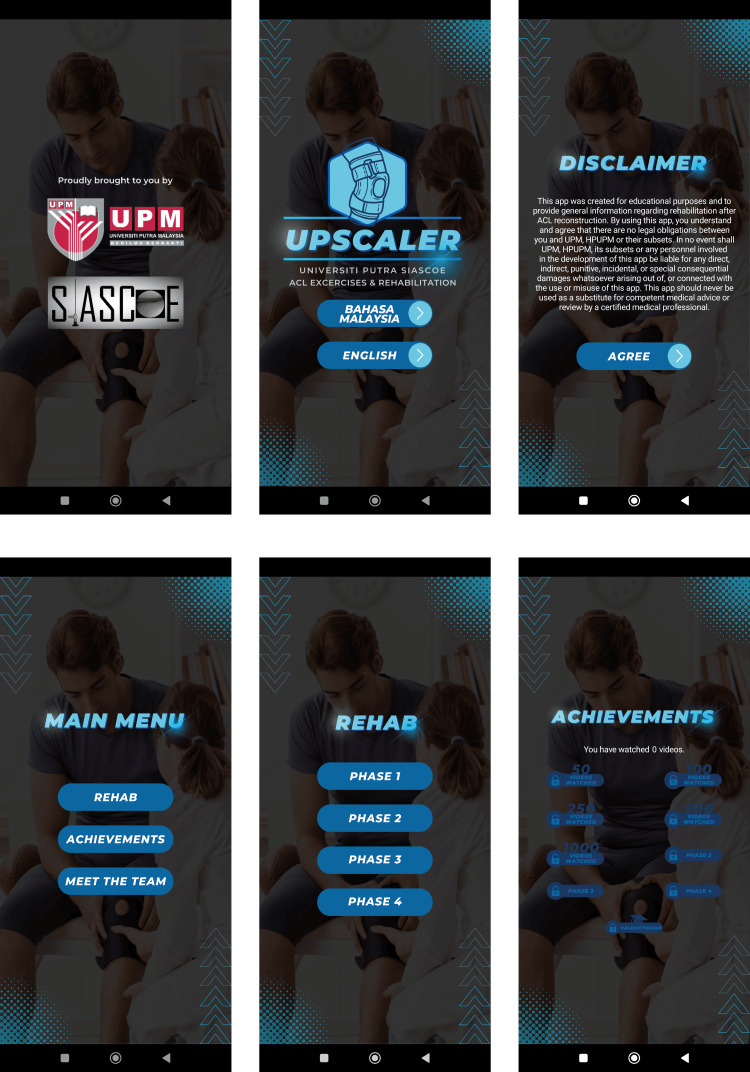
UPSCALER screenshots. UPSCALER: Universiti Putra SIASCOE (Sports Injury and Arthroscopic Surgery Center of Excellence) ACL (Anterior Cruciate Ligament) Exercises & Rehabilitation.
